# Comparative effects of EMG-driven robot-assisted therapy versus task-oriented training on motor and daily function in patients with stroke: a randomized cross-over trial

**DOI:** 10.1186/s12984-021-00961-w

**Published:** 2022-01-16

**Authors:** Yen-Wei Chen, Wei-Chi Chiang, Chia-Ling Chang, Shih-Ming Lo, Ching-Yi Wu

**Affiliations:** 1 Department of Occupational Therapy and Graduate Institute of Behavioral Sciences, College of Medicine, Chang Gung University, No.259, Wenhua 1st Rd., Guishan Dist., Taoyuan City, 33302 Taiwan; 2 Department of Occupational Therapy, I-Shou University, No.8, Yida Rd., Yanchao Dist., Kaohsiung City, 82445 Taiwan; 3 Chang Gung Memorial Hospital, Taipei Branch, No.199, Tung Hwa North Road, Taipei City, 10507 Taiwan; 4 Healthy Aging Research Center, Chang Gung University, Taoyüan, Taiwan; 5 Department of Physical Medicine and Rehabilitation, Chang Gung Memorial Hospital at Linkou, Taoyüan, Taiwan

**Keywords:** Stroke, Robot-assisted therapy, Upper extremity, Rehabilitation

## Abstract

**Background:**

Robot-assisted hand training has shown positive effects on promoting neuromuscular control. Since both robot-assisted therapy and task-oriented training are often used in post-stroke rehabilitation, we raised the question of whether two interventions engender differential effects in different domains.

**Methods:**

The study was conducted using a randomized, two-period crossover design. Twenty-four chronic stroke survivors received a 12-session robot-assisted intervention followed by a 12-session task-oriented intervention or vice versa. A 1-month washout period between each intervention was implemented. Outcome measures were evaluated before the intervention, after the first 12-session intervention, and after the second 12-session intervention. Clinical assessments included Fugl-Meyer Assessment for Upper Extremity, Wolf Motor Function Test, Action Research Arm Test and Motor Activity Log.

**Results:**

Our findings suggested that EMG-driven robot-assisted therapy was as effective as task-oriented training in terms of improving upper limbs functional performance in activity domain, and robot-assisted therapy was more effective in improving movement duration during functional tasks. Task-oriented training showed better improvement in body function domain and activity and participation domain, especially in improving spontaneous use of affected arm during daily activities.

**Conclusions:**

Both intervention protocol had their own advantages in different domains, and robot-assisted therapy may save manpower and be considered as an alternative intervention to task-oriented training. Combining the two approaches could yield results greater than either alone, which awaits further study.

*Trial registration*: ClinicalTrials.gov Identifier: NCT03624153. Registered on 9th August 2018, https://clinicaltrials.gov/ct2/show/NCT03624153.

## Background

Upper limbs dysfunction is a common sequela of stroke. Dysfunction in upper limbs is a combination of muscle weakness, poor dexterity, incoordination, sensory loss and abnormal motor synergies, which impairs the performance of activities of daily living (ADLs). Hand function accounted for most of delicate movements in daily activities [1, 2] and deficits in hand movements seriously influence performance of a variety of daily tasks. A decline in functional independence after stroke not only leads to the deterioration in quality of life, but also places heavy pressure on caregivers. Thus, restoration of upper limbs function is identified as a top priority for stroke patients, ranked by stroke patients, caregivers and medical professionals [3, 4].

Restoration of motor function after stroke requires intense and massed practice for desired motor skills [3, 5, 6]. Among several contemporary approaches, robot-assisted therapy has gained acceptance in upper limbs rehabilitation [6–8]. Robot-assisted therapy provides patients with intense, repetitive practice and precise motion guidance which could promote neuromuscular control and reverse learned nonuse phenomenon [9]. Many rehabilitation robots for the upper limbs are available in the market and there is an increasing trend of using exoskeleton robots [10, 11]. Exoskeleton robots are wearable devices with joints and links which correspond to those of the human body [12]. The advantage of exoskeleton robots is that they provide precise control over multiple joints and allow training at selective joints. Control strategy is a major aspect that enables the robot to provide assisted movement [13]. During active-assisted training mode, the robot detects the patient’s intention to move through the sensor and trigger the assisted movement that allow the patient’s hand actively interacts with the robot. The sensors that integrated into the robots can be classified into physical sensors and bioelectrical sensors [14]. Physical sensors such as torque sensors and position sensors are the most used sensors in rehabilitation robots for the upper limbs [15]. However, physical sensors require some degree of volitional movement to trigger, and those devices might not benefit patients who are unable to generate sufficient force to trigger robots. In contrast to physical sensors, bioelectrical sensors such as electromyography (EMG) sensors can detect patients’ voluntary muscle activation in real time and triggered the robot-assisted movement, which could be beneficial in a broader range of patients. The aim of this study was to examine the therapeutic effects of robot-assisted therapy in neurorehabilitation using an EMG-driven exoskeleton hand robot.

EMG-driven hand robot has shown to increase voluntary motor control, decrease muscle spasticity and improve upper limbs motor function [16–18], but there were limited studies mainly compared the effects between EMG-driven robot-assisted therapy and task-oriented training in patients with stroke. Task-oriented training is a client-centered and functional-based intervention, which has demonstrated effectiveness on motor recovery after stroke [19] and been commonly incorporated into routine practice in occupational therapy for stroke rehabilitation [20, 21]. In this study, we intended to examine whether EMG-driven robot-assisted therapy and task-oriented training engenders differential effects in different domains.

## Methods

### Study design and participants

**Fig. 1:**
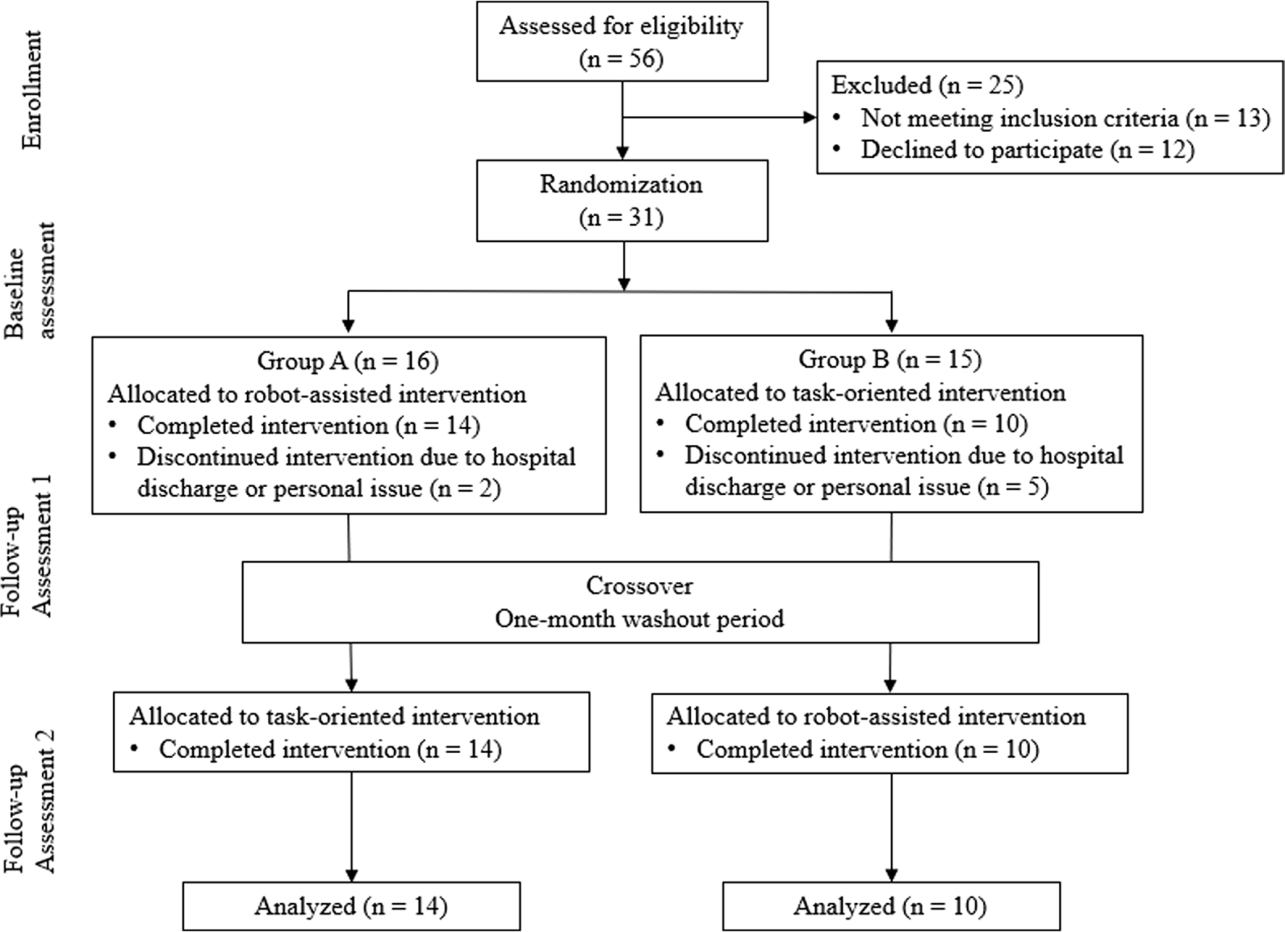
Flow diagram illustrating the flow of participants through each stage of the study

This study was performed in a randomized, single-blind, two-period crossover design to compare the efficacy of robot-assisted intervention with task-oriented intervention in chronic stroke patients. The Institutional Review Board for Human Studies approved the protocol, and all participants provided written informed consent before participating. Participants were recruited from medical centers in Taiwan. The inclusion criteria included: (1) unilateral stroke ≥ 3 months prior to study enrollment; (2) Fugl-Meyer Assessment for Upper Extremity (FMA-UE) score < 60; (3) without excessive spasticity in any of the UE joint (modified Ashworth scale ≤ 3); (4) Mini Mental State Exam (MMSE) score > 24, indicating no serious cognitive impairment; and (5) between the ages of 20 and 75 years. The exclusion criteria included: (1) histories of other neurological diseases such as dementia and peripheral polyneuropathy; (2) difficulties in following and understanding instructions such as global aphasia; (3) enroll in other rehabilitation or drug studies simultaneously; (4) receiving Botulinum toxin injections within 3 months. The research design and flow process are shown in Fig. 1.

### Equipment

The Hand of Hope (HOH) robotic hand system (Rehab-Robotics Co. Ltd, Hongkong, China) was used in this study. HOH is an exoskeleton type of robot which is worn on the dorsal side of the impaired hand with two surface EMG sensors attached to the extensor digitorum and flexor digitorum superficialis. The system detects EMG signals during voluntary muscle contraction and provides patients with real-time visual feedback to identify the level of motor unit recruitment. Training modes of HOH include “continuous passive motion”, “EMG biofeedback—trigger and go”, “EMG biofeedback—trigger and maintain”, and "interactive games". In continuous passive motion mode, the robot provides passive range of motion to the hand involving all fingers or three fingers (thumb, index finger and middle finger). In EMG biofeedback—trigger and go mode, the robot guides the patient to complete the hand motion in full range once the EMG signal exceeds the predetermined threshold. In EMG biofeedback—trigger and maintain mode, the patient is required to keep generating EMG signals above the predetermined threshold to activate the robot-assisted hand motion. The primary difference between the two EMG biofeedback modes is the amount of assistance provided by the robot. In interactive gaming mode, the robot provides patients with video games, which incorporate hand and arm movements with interactive games. The system comes with a series of interactive games and therapists can choose the level of difficulty in games based on patients’ upper limb functional status.

### Intervention protocol

Participants were randomly assigned to group A or group B using a computer-generated list. Participants in group A received 12 sessions of robot-assisted intervention first, followed by a 1-month washout period, and then received 12 sessions of task-oriented intervention. Participants in group B received 12 sessions of task-oriented intervention first, followed by a 1-month washout period, and then received 12 sessions of robot-assisted intervention. All interventions were conducted by a certified occupational therapist. The flow of the study was illustrated in Fig. 1.

#### Robotic-assisted intervention protocol

Participants received the 12-session robot-assisted intervention 3 sessions a week for 4 consecutive weeks. Each session consisted of 20-min continuous passive motion, 20-min active motion practice, and 30-min interactive gaming practice. Before each intervention session, participants were instructed to perform maximum voluntary isometric contraction (MVIC) of hand grasping and hand opening with HOH system. The system detected the EMG levels at MVIC and the threshold for EMG trigger during biofeedback mode was determined by 50% of MVIC.

In continuous passive motion practice, the robot provided passive grasp and release motion with all fingers for 10 min, and passive pinch and release motion with thumb, index finger and middle finger for 10 min. In active motion practice, participants received 10-min biofeedback training to practice grasp and release motion and 10-min biofeedback training to practice pinch and release motion in either “trigger and go” mode or “trigger and maintain” mode based on patients’ motor status. In interactive gaming practice, the therapist selected three activities that incorporate hand and arm movements with interactive video games.

#### Task-oriented intervention protocol

Participants received the 12-session task-oriented intervention 3 sessions a week for 4 consecutive weeks. Each session consisted of 20-min warm up including range of motion exercise and strengthening exercise followed by 50-min task-oriented training for activities of daily living under the supervision of a senior occupational therapist.

### Outcome measurements

The clinical assessments we used to evaluate therapeutic effects of robot-assisted intervention and task-oriented intervention focused on domains of the International Classification of Functioning, Disability and Health (ICF) framework: (1) body function and (2) activity and participation. Clinical assessments included Fugl-Meyer Assessment for Upper Extremity (FMA-UE) that falls within the body function domain [22], Wolf Motor Function Test (WMFT) and Action Research Arm Test (ARAT) that falls within the activity domain [22], and Motor Activity Log (MAL) that falls within the activity and participation domains [22, 23]. Participants were assessed before the intervention (baseline assessment), around 2 weeks after the first 12-session intervention (follow-up assessment 1), and after the second 12-session intervention (follow-up assessment 2) [24]. All participants were assessed by a certified occupational therapist who was unaware of the group to which the participant had been allocated.

#### Domain of body function

Fugl-Meyer Assessment for Upper Extremity (FMA-UE): The FMA-UE falls within the body function domain of ICF framework, which includes 33 items assessing movements, reflexes, and coordination of upper limbs. Each item is measured on a 3-point ordinal scale and the total score ranges from 0 to 66 [25]. A higher score indicates better motor function. The reliability and validity of the Fugl-Meyer Assessment are well established [1, 26].

#### Domain of activity and participation

Wolf Motor Function Test (WMFT): The WMFT falls within the activity domain of ICF framework, which assesses upper extremity motor ability by measuring the performance time (WMFT-Time) and functional ability rating scale (WMFT-FAS) in required task. Participants were timed and rated by using a 6-point ordinal scale. The WMFT is valid and reliable on assessing motor function in stroke patients [27, 28].

The Action Research Arm Test (ARAT): The ARAT falls within the activity domain of ICF framework, which assess upper limbs performance in stroke recovery. It consists of 19 items categorized into four subscales: grasp, grip, pinch, and gross movement. Performance on each item is rated on a 4-point ordinal scale and a higher score indicates better performance. Reliability and validity of the ARAT have been well established [29].

Motor Activity Log (MAL): The MAL falls within the activity and participation domains of ICF framework. It is a semi-structured interview for stroke patients to assess the amount of use (MAL-AOU) and quality of movement (MAL-QOM) of their affected arm and hand during 30 activities of daily living. The score of each activity ranges from 0 to 5, and higher scores represent more frequently used or higher quality of movement. The MAL has good validity, reliability and responsiveness in patients with stroke. [30–32]

### Statistical analysis

We used *Chi-*square tests and independent *t*-tests to compare participants’ baseline characteristics between group A and B. Before we examined the treatment effects between interventions, we conducted a preliminary test to examine if the carryover effect is negligible [33]. We calculated the sum of the measured scores post-intervention (follow-up assessment 1 and 2) for each participant and compared the means between two sequences (group A and group B) by independent t-tests. A non-significant result suggested that carryover effects should be null or negligible.

We used repeated measures ANOVA to examine treatment effects between interventions (robotic-assisted intervention vs. task-oriented intervention). We defined “intervention” as within-subject factor and “group” as between-subject factor. For all calculations, a significance level at α = 0.05 was used. Post hoc paired t tests with Bonferroni correction were applied. If the interaction effect between intervention and group was significant, we reconducted repeated measures ANOVA for two groups separately to examine the intervention effect. All tests were executed using the SPSS software version 25 (International Business Machines Corp., Armonk, NY).

## Results

### Demographic characteristics of both groups

We screened 56 patients for eligibility. Thirty-one of them met the inclusion criteria and were randomly assigned to two groups. During the first intervention period, seven participants withdrew from the study and they were excluded from data analysis. Descriptive characteristics of participants are presented in Table 1. The two groups did not differ significantly in terms of participants’ demographic and clinical characteristics, except for age. Participants in group A were significantly younger than those in group B (54.58 ± 10.98 years vs. 64.98 ± 8.22 years; *p* = .02).

**Table 1:** Demographic characteristics and clinical background of participants

Variables	Group A (N = 14)	Group B (N = 10)	*p* value
Gender (male/female)	10/4	9/1	.28
Affected side (R/L)	8/6	7/3	.42
Age (years), mean ± SD	54.58 ± 10.98	64.98 ± 8.22	.02
Time since stroke (months), mean ± SD	37.07 ± 34.39	59.8 ± 43.34	.17
FMA-UE, mean ± SD	33 ± 8.53	36.4 ± 10.1	.38
MMSE, mean ± SD	28 ± 1.52	26.8 ± 2.86	.19

*FMA-UE* Fugl-Meyer Assessment for upper extremity; *MMSE* Mini Mental State Exam

### Carry-over effects

The first step of our analysis was to examine carryover effects between both intervention periods. Statistical analysis revealed no carryover effects for all outcome parameters (FMA-UE: *p* = .86, FMA-proximal: *p* = .86, FMA-distal: *p* = .65, WMFT-Time: *p* = .27, WMFT-FAS: *p* = .20, MAL-AOU: *p* = .53, MAL-QOM: *p* = .93, ARAT: *p* = .41).

### Domain of body function

The mean and standard deviation for clinical outcome measures were shown in Table 2. Results of inferential statistics were shown in Table 3. Results of FMA-UE revealed a statistically significant interaction effect between intervention and group, F(2,44) = 4.64, p = .015. Therefore, we tested the effect of intervention for group A and group B separately. In robotic-assisted intervention first group, we found a statistically significant effect of intervention, F(2,26) = 8.37, p = .002. Pairwise comparisons revealed that participants in this group significantly improved upper limb motor function after receiving robot-assisted intervention and after receiving task-oriented intervention (p = .031, .013 respectively). The improvements were not significantly different between the two interventions (p = .91). In task-oriented intervention first group, we found a statistically significant effect of intervention, F(2,18) = 5.29, p = .016. Pairwise comparisons revealed that participants in this group significantly improved upper limb motor function after task-oriented intervention (p = .018), but not after receiving robot-assisted intervention (p = .816).

**Table 2:** Descriptive statistics for clinical outcome measures

Outcome measure	Baseline		End of robot-assisted intervention		End of conventional intervention	
	Group A	Group B	Group A	Group B	Group A	Group B
FMA-UE	33 (8.53)	36.4 (10.1)	35.64 (9.3)	34.7 (11.02)	36.43 (9.53)	38.8 (10.32)
WMFT-Time	14.85 (6.19)	12.09 (6.5)	12.52 (5.92)	9.07 (4.51)	12.41 (6.21)	11.05 (6.03)
WMFT-FAS	2.51 (.44)	2.65 (.71)	2.63 (.58)	2.83 (.77)	2.75 (.7)	3.31 (.99)
MAL-AOU	1.15 (.82)	.97 (1.13)	1.33(.82)	1.09 (1.15)	1.58 (1.04)	1.25 (1.27)
MAL-QOM	.76 (.48)	.78 (.95)	.86(.56)	1.12 (1.07)	1.08 (.76)	.89 (.94)
ARAT	14.5 (9.24)	18.8 (12.13)	16(9.03)	22.4 (10.34)	20.07 (13.98)	21.9 (15.2)

Data are presented as the mean (SD)

*FMA_UE* upper limb subtest of the Fugl-Meyer assessment, *WMFT-Time* performance time of the Wolf Motor Function Test, *WMFT-FAS* functional ability scale of the Wolf Motor Function Test, *MAL-AOU* Motor Activity Log Amount of use score, *MAL-QOM* Motor Activity Log Quality of movement score, *ARAT* Action Research Arm Test

**Table 3:** Inferential statistics for outcome measures

Outcome measure	Effect	*df*	F	*p*
FMA-UE	Within subjects			
	Intervention	2	8.82	.001
	Intervention × group	2	4.64	.015
	Error	44		
	Between subjects			
	Group	1	.17	.686
	Error	22		
WMFT-Time	Within subjects			
	Intervention	2	4.59	.015
	Intervention × group	2	.71	.499
	Error	44		
	Between subjects			
	Group	1	1.26	.273
	Error	22		
WMFT-FAS	Within subjects			
	Intervention	2	6.89	.002
	Intervention × group	2	1.67	.2
	Error	44		
	Between subjects			
	Group	1	1.5	.233
	Error	22		
MAL-AOU	Within subjects			
	Intervention	2	5.9	.005
	Intervention × group	2	.29	.746
	Error	44		
	Between subjects			
	Group	1	.38	.54
	Error	22		
MAL-QOM	Within subjects			
	Intervention	2	5.25	.009
	Intervention × group	2	4.33	.02
	Error	44		
	Between subjects			
	Group	1	.01	.919
	Error	22		
ARAT	Within subjects			
	Intervention	2	5.48	.007
	Intervention × group	2	1.51	.232
	Error	44		
	Between subjects			
	Group	1	.82	.375
	Error	22		

Main effects of within-subject factor (intervention) and between-subjects factor (group), along with interactions

### Domain of activity and participation

Results of WMFT-Time showed no statistically significant interaction between groups and intervention, p = .49 (see Table 3). For the main effect, there was a statistically significant effect of intervention, F(2,44) = 4.59, p = .02, but no statistically significant effect of group (p = .27). Pairwise comparisons revealed that participants significantly improved their performance time for completing the tasks after receiving robot-assisted intervention (p = .004), but no significant improvement was found after receiving task-oriented intervention (p = .21).

As for the results of WMFT-FAS, there was no statistically significant interaction between groups and intervention (p = .20). For the main effect, we found a statistically significant effect of intervention F(2,44) = 6.89, p = .002, but there was no statistically significant effect of group (p = .23). Pairwise comparisons revealed that participants significantly improved their functional ability after receiving robot-assisted intervention and after receiving task-oriented intervention (p = .026, .031 respectively). Both interventions showed positive effect in improving functional ability, and the improvements were not significantly different between the two interventions (p = .12).

The ARAT results showed no statistically significant interaction between groups and intervention (p = .35). For the main effect, there was a statistically significant effect of intervention, F(2,44) = 5.48, p = .007, but there was no statistically significant effect of group (p = .38). Pairwise comparisons revealed that participants significantly improved their upper limbs performance after receiving robot-assisted intervention and after receiving task-oriented intervention (p = .03, .001 respectively). Both interventions were effective in improving upper limbs functional performance, and the improvements were not significantly different between the two interventions (p = .87).

Results of MAL-AOU showed no statistically significant interaction effect between group and intervention (p = .75). For the main effect, there was a statistically significant effect of intervention, F(2,44) = 5.9, p = .005, but there was no statistically significant effect of group (p = .54). Pairwise comparisons revealed that participants significantly increased the affected arm use after receiving task-oriented intervention (p = .014), but no significant difference was found after receiving robot-assisted intervention (p = .57).

Results of MAL-QOM revealed a statistically significant interaction effect between intervention and group, F(2,44) = 4.33, p = .019. Therefore, we tested the effect of intervention for two groups separately. In robotic-assisted intervention first group, we found a statistically significant effect of intervention, F(2,26) = 4.84, p = .016. Pairwise comparisons revealed that participants in this group significantly improve movement quality after receiving task-oriented intervention (p = .03), but no significant improvement was found after receiving robot-assisted intervention (p = .24). In task-oriented intervention first group, we found a statistically significant effect of intervention, F(2,18) = 5.26, p = .016. Pairwise comparisons revealed that participants in this group significantly improve movement quality after receiving robot-assisted intervention (p = .03), but no significant improvement was found after receiving task-oriented intervention (p = .22).

## Discussion

In this study, we used a randomized, two-period crossover design to examine whether robot-assisted therapy and task-oriented training for chronic stroke survivors engender differential effects in different domains. Clinical assessments that focus on domains of body function and domain of activity and participation were used to examine intervention effects of each intervention protocol. Our findings indicated that both EMG-driven robot-assisted therapy and task-oriented training were effective in improving upper extremity function in patients with stroke, and each intervention protocol had its own advantages in different domains. Robot-assisted intervention showed better improvement in activity domain, especially in performance time, and task-oriented intervention showed better improvement in body function domain.

In body function domain, results of FMA-UE showed that participants in two groups responded differently to different intervention. Robot-assisted intervention significantly improved participants’ motor function in robot-assisted intervention first group, and task-oriented intervention significantly improved participants’ motor function in both groups. The participants, who received robot-assisted intervention first, might acquire basic motor skills first because robot-assisted intervention offered repetitive practice and consistent movement trajectory to patients, which are fundamental for learning basic motor skills. After participants obtained the basic motor skills in robot-assisted intervention, they practiced functional tasks further enhancing motor skills via a variety of functional practice in task-oriented intervention. In contrast, participants who received task-oriented intervention first might directly improve motor skills through practice the functional use of the affected arm in terms of task-oriented training. Therefore, the robot-assisted intervention following the task-oriented intervention training might play a minor role to reduce motor impairments. These findings suggested task-oriented training might be important for reducing motor impairments, and robot-assisted intervention might assist patients to regain the motor skills at the beginning stage and save, at least in part, the manpower.

In activity and participation domains, both intervention protocols showed comparably positive effect on activity performance, measured by WMFT-FAS and ARAT. However, we found that robot-assisted intervention was superior to task-oriented intervention in terms of improving performance time of functional tasks. The WMFT-Time scores revealed that participants significantly spent less time to complete the tasks after receiving robot-assisted intervention, but the performance time did not improve after receiving task-oriented intervention. One explanation is that EMG-driven robot could assist patients in precisely facilitating desired muscle groups during training and improving coordination of agonist and antagonist muscles around joints. Participants with better muscular control could improve their movement time during functional tasks. Another explanation is that task-oriented intervention focuses on the completion of ADLs with less attention paid towards movement speed, while robot-assisted intervention incorporates movement speed into the training program. Therefore, participants could improve their movement time during functional tasks after receiving robot-assisted intervention.

Results of MAL-AOU revealed that task-oriented intervention had more benefits in terms of improving spontaneous use of affected upper limb during daily activities, compared with robot-assisted intervention. Regarding our intervention protocols, task-oriented intervention mainly focused on practice of a variety of task-oriented functional activities while robot-assisted intervention focused on movement training in distal parts of upper limb. Although the robot-assisted intervention provided interactive gaming mode in HOH robot system, the variety of games is limited and movements in those games were primarily performed in a two-dimensional plane. As a result, participants might easily transfer the skills acquired in task-oriented intervention to their daily activities and spontaneously make use of their affected upper limbs in their housing environment. Results of MAL-QOM showed that participants reported better movement quality during daily activities after receiving the second intervention protocol, regardless of their groups. We suspected that the improvement related to time-dependent changes in motor recovery. Since the MAL-QOM primarily assessed the involvement of the affected arm during functional activities which might need more practice to improve, participants exhibited better involvement of the affected arm during functional activities after receiving the second intervention protocol.

This study has some limitations. First, cross-over studies usually require longer duration than parallel-group studies. Prolonged study duration and repeated assessments could lead to an increase in dropout rates. In this study, seven participants dropped out during the first intervention period that resulted in a reduction of the sample size and unequal number of participants in each group. There were fewer participants in group B (task-oriented intervention-first group) in our analysis, which could influence the results. Second, participants were significantly older in task-oriented intervention first group. Since we did not find significant differences in impairment level at baseline measurement between two groups, the impact of age that moderates the effect of rehabilitation training may be limited. Third, participants were allowed to practice upper-limb movement or participate in functional activities using affected arms during washout period. Although a 1-month washout period was implemented and statistical analysis showed that no carryover effect was present, it’s possible that new acquired motor skills and control sequences could be retained after the washout period. Fourth, the feature of the robotic device might influence the effect of robot-assisted intervention. For example, an innovative exoskeleton robotic hand with neuromuscular electrical stimulation function has been developed to facilitate muscular coordination [18]. The combined treatment with robot and neuromuscular electrical stimulation could augment the effect of robot-assisted intervention. Although a growing number of robotic devices have been developed, only a few have been commercialized. Therefore, we chose the EMG-driven exoskeleton robotic hand available on the market to conduct this study. Future studies are needed to explore the alternative intervention protocol to improve the effectiveness of robot-assisted intervention. Lastly, we used clinical assessments to measure functional improvement after interventions and these clinical assessments could lack sensitivity to detect minor changes in motor performance due to the nature of ordinal scales. Since the goal of EMG-driven robot-assisted therapy is to improve awareness of muscle control and to educate appropriate muscle groups to produce movements, future studies are needed to investigate intervention effects on motor performance and motor unit recruitment using instrumental assessments, such as electromyography and kinematic analysis.

## Conclusion

Robot-assisted therapy offers the advantage of manpower saving and delivering intensive training in neurorehabilitation. The purpose of the study was to examine the effects of EMG-driven robot-assisted therapy on motor function in patients with stroke, compared with task-oriented training. Our findings suggested that EMG-driven robot-assisted therapy is as effective as task-oriented training in terms of improving upper limbs functional performance in activity domain, and robot-assisted therapy is more effective in improving movement duration during functional tasks. While EMG-driven robot-assisted therapy is not as effective as task-oriented training in body function domain and activity and participation domain, we hypothesized that combining two interventions would yield greater intervention effects in patients with stroke. Future research should be conducted to determine the effects of combined intervention protocol in patients with stroke.

## Data Availability

The data set used and/or analyzed during the current study is available from the corresponding author upon reasonable request.

## Abbreviations

ADLs: Activities of daily living
ARAT: Action Research Arm Test
EMG: Electromyography
FMA_UE: Fugl-Meyer Assessment for Upper Extremity
HOH: Hand of Hope
ICF: International Classification of Functioning, Disability and Health
MAL: Motor Activity Log
MAL-AOU: Motor Activity Log Amount of use score
MAL-QOM: Motor Activity Log Quality of movement score
MMSE: Mini Mental State Exam
MVIC: Maximum voluntary isometric contraction
WMFT: Wolf Motor Function Test
WMFT-FAS: Functional ability scale of the Wolf Motor Function Test
WMFT-Time: Performance time of the Wolf Motor Function Test

## References

[1] Gladstone DJ, Danells CJ, Black SE (2002). The Fugl-Meyer Assessment of motor recovery after stroke: a critical review of its measurement properties. Neurorehabil Neural Repair.

[2] Simpson LA, Eng JJ (2013). Functional recovery following stroke: capturing changes in upper-extremity function. Neurorehabil Neural Repair.

[3] Chang WH, Kim Y-H (2013). Robot-assisted therapy in stroke rehabilitation. J Stroke.

[4] Huang P-C, Hsieh Y-W, Wang C-M, Wu C-Y, Huang S-C, Lin K-C (2014). Predictors of motor, daily function, and quality-of-life improvements after upper-extremity robot-assisted rehabilitation in stroke. Am J Occup Ther.

[5] Duret C, Courtial O, Grosmaire A-G, Hutin E (2015). Use of a robotic device for the rehabilitation of severe upper limb paresis in subacute stroke: exploration of patient/robot interactions and the motor recovery process. Biomed Res Int.

[6] Volpe BT, Lynch D, Rykman-Berland A, Ferraro M, Galgano M, Hogan N (2008). Intensive sensorimotor arm training mediated by therapist or robot improves hemiparesis in patients with chronic stroke. Neurorehabil Neural Repair.

[7] Conroy SS, Whitall J, Dipietro L, Jones-Lush LM, Zhan M, Finley MA (2011). Effect of gravity on robot-assisted motor training after chronic stroke: a randomized trial. Arch Phys Med Rehabil.

[8] Kwakkel G, Kollen BJ, Krebs HI (2008). Effects of robot-assisted therapy on upper limb recovery after stroke: a systematic review. Neurorehabil Neural Repair.

[9] Susanto EA, Tong RK, Ockenfeld C, Ho NS (2015). Efficacy of robot-assisted fingers training in chronic stroke survivors: a pilot randomized-controlled trial. J NeuroEng Rehabil.

[10] Zhang K, Chen X, Liu F, Tang H, Wang J, Wen W (2018). System framework of robotics in upper limb rehabilitation on poststroke motor recovery. Behav Neurol.

[11] Aggogeri F, Mikolajczyk T, O’Kane J (2019). Robotics for rehabilitation of hand movement in stroke survivors. Adv Mech Eng.

[12] Maciejasz P, Eschweiler J, Gerlach-Hahn K, Jansen-Troy A, Leonhardt S (2014). A survey on robotic devices for upper limb rehabilitation. J NeuroEng Rehabil.

[13] Sandoval-Gonzalez O, Jacinto-Villegas J, Herrera-Aguilar I, Portillo-Rodiguez O, Tripicchio P, Hernandez-Ramos M (2016). Design and development of a hand exoskeleton robot for active and passive rehabilitation. Int J Adv Rob Syst.

[14] Yue Z, Zhang X, Wang J (2017). Hand rehabilitation robotics on poststroke motor recovery. Behav Neurol.

[15] Bos RA, Haarman CJW, Stortelder T, Nizamis K, Herder JL, Stienen AHA (2016). A structured overview of trends and technologies used in dynamic hand orthoses. J NeuroEng Rehabil.

[16] Ho NSK, Tong KY, Hu XL, Fung KL, Wei XJ, Rong W, et al. An EMG-driven exoskeleton hand robotic training device on chronic stroke subjects: task training system for stroke rehabilitation. In: 2011 IEEE international conference on rehabilitation robotics [Internet]. Zurich: IEEE; 2011 [cited 2020 Jun 5]. p. 1–5. Available from: http://ieeexplore.ieee.org/document/5975340/. 10.1109/ICORR.2011.5975340 22275545

[17] Hu XL, Tong KY, Wei XJ, Rong W, Susanto EA, Ho SK (2013). The effects of post-stroke upper-limb training with an electromyography (EMG)-driven hand robot. J Electromyogr Kinesiol.

[18] Nam C, Zhang B, Chow T, Ye F, Huang Y, Guo Z (2021). Home-based self-help telerehabilitation of the upper limb assisted by an electromyography-driven wrist/hand exoneuromusculoskeleton after stroke. J NeuroEng Rehabil.

[19] Bosch J, O’Donnell MJ, Barreca S, Thabane L, Wishart L (2014). Does task-oriented practice improve upper extremity motor recovery after stroke? A systematic review. ISRN Stroke.

[20] Almhdawi KA, Mathiowetz VG, White M, delMas RC (2016). Efficacy of occupational therapy task-oriented approach in upper extremity post-stroke rehabilitation. Occup Ther Int.

[21] Hubbard IJ, Parsons MW, Neilson C, Carey LM (2009). Task-specific training: evidence for and translation to clinical practice. Occup Ther Int.

[22] Santisteban L, Térémetz M, Bleton J-P, Baron J-C, Maier MA, Lindberg PG (2016). Upper limb outcome measures used in stroke rehabilitation studies: a systematic literature review. PLoS ONE.

[23] Tse T, Douglas J, Lentin P, Carey L (2013). Measuring participation after stroke: a review of frequently used tools. Arch Phys Med Rehabil.

[24] Sczesny-Kaiser M, Trost R, Aach M, Schildhauer TA, Schwenkreis P, Tegenthoff M (2019). A randomized and controlled crossover study investigating the improvement of walking and posture functions in chronic stroke patients using HAL exoskeleton—the HALESTRO Study (HAL-Exoskeleton STROke Study). Front Neurosci.

[25] Fugl-Meyer AR, Jääskö L, Leyman I, Olsson S, Steglind S (1975). The post-stroke hemiplegic patient 1. A. method for evaluation of physical performance. Scand J Rehabil Med.

[26] Platz T, Pinkowski C, van Wijck F, Kim I-H, di Bella P, Johnson G (2005). Reliability and validity of arm function assessment with standardized guidelines for the Fugl-Meyer Test, Action Research Arm Test and Box and Block Test: a multicentre study. Clin Rehabil.

[27] Morris DM, Uswatte G, Crago JE, Cook EW, Taub E (2001). The reliability of the wolf motor function test for assessing upper extremity function after stroke. Arch Phys Med Rehabil.

[28] Wolf SL, Catlin PA, Ellis M, Archer AL, Morgan B, Piacentino A (2001). Assessing Wolf Motor Function Test as outcome measure for research in patients after stroke. Stroke.

[29] Van der Lee JH, De Groot V, Beckerman H, Wagenaar RC, Lankhorst GJ, Bouter LM (2001). The intra- and interrater reliability of the action research arm test: a practical test of upper extremity function in patients with stroke. Arch Phys Med Rehabil.

[30] Uswatte G, Taub E, Morris D, Light K, Thompson PA (2006). The Motor Activity Log-28: assessing daily use of the hemiparetic arm after stroke. Neurology.

[31] Uswatte G, Taub E, Morris D, Vignolo M, McCulloch K (2005). Reliability and validity of the upper-extremity Motor Activity Log-14 for measuring real-world arm use. Stroke.

[32] van der Lee JH, Beckerman H, Knol DL, de Vet HCW, Bouter LM (2004). Clinimetric properties of the motor activity log for the assessment of arm use in hemiparetic patients. Stroke.

[33] Wellek S, Blettner M (2012). On the proper use of the crossover design in clinical trials. Dtsch Arztebl Int.

